# Improvement of aphasia deficits following traumatic intracranial hemorrhage: a case report

**DOI:** 10.25122/jml-2026-0072

**Published:** 2026-07

**Authors:** Junhyun Choi

**Affiliations:** 1 Department of Rehabilitation Medicine, St. Carollo Hospital, Suncheon, Republic of Korea

**Keywords:** traumatic intracranial hemorrhage, aphasia, Cerebrolysin, speech-language therapy, case report

## Abstract

Aphasia is a common and disabling consequence of dominant-hemisphere brain injury, and the pharmacological options after traumatic intracranial hemorrhage are limited. A 69-year-old man developed severe aphasia after traumatic intracranial hemorrhage. On day 7 after traumatic brain injury (TBI), he was transferred for inpatient rehabilitation, where the Korean version of the Western Aphasia Battery (K-WAB) showed marked language impairment, with an Aphasia Quotient (AQ) of 66.2 (scale 0–100; higher scores indicate better language function). He received intravenous Cerebrolysin, 30 mL/day infused over 60 minutes, for 21 consecutive days, together with daily speech-language therapy. By day 28, the AQ had risen to 97.1, with gains in both expressive and receptive language, and no adverse events were recorded. Spontaneous subacute recovery and the concurrent speech-language therapy may account for the change, and a single patient cannot establish an effect; the time course and the size of the change nonetheless raise the possibility of an adjunctive neurorestorative contribution from Cerebrolysin in post-traumatic hemorrhagic aphasia. Controlled studies in TBI populations are needed.

## Introduction

Aphasia is a common and disabling consequence of dominant-hemisphere brain injury, including hemorrhagic contusion [1]. Compared with ischemic and hemorrhagic events, pharmacologic strategies to enhance neurorecovery after traumatic intracranial hemorrhage remain relatively limited [2]. Currently, there are no universally accepted therapeutic strategies for acute TBI neurorecovery; however, interventions such as amantadine and methylphenidate are occasionally utilized off-labelin clinical practice to promote arousal and cognitive processing, albeit with mixed efficacy [3]. Cerebrolysin, a neuropeptide preparation with neurotrophic properties, has shown benefits in neuroprotection, synaptic plasticity, and functional recovery, particularly in ischemic events and certain types of spontaneous intracerebral hemorrhage (ICH) [4,5]. A recent randomized controlled trial reported a therapeutic effect of Cerebrolysin in post-ischemic aphasia [6], but clinical evidence in post-traumatic aphasia remains scarce. We report a case of substantial language recovery after a 21-day course of Cerebrolysin combined with conventional speech-language therapy (SLT) in a patient with left traumatic frontal-temporal ICH and subarachnoid hemorrhage (SAH). To ensure transparency and completeness, this manuscript was prepared in accordance with the CARE guidelines [7].

## Case presentation

### Patient information

**Figure 1:**
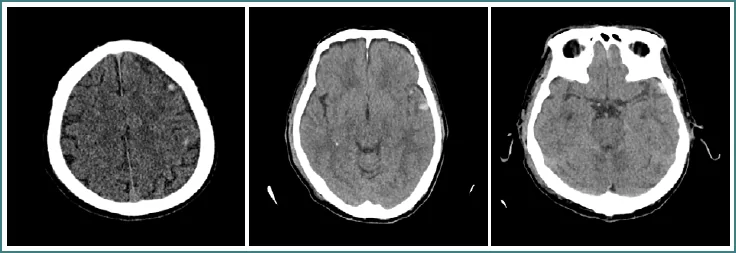
Brain CT on admission. Multiple parallel axial slices highlight the multifocal hemorrhagic contusions in the left frontal and temporal lobes.

A 69-year-old male with no significant prior medical history was found collapsed at his workplace around lunchtime, having reported to work as usual that morning. Heteroanamnesis obtained from coworkers and emergency responders indicated a presumed mechanical fall resulting in head trauma. He first presented to the emergency department of another hospital, where brain CT showed hemorrhagic contusion of the left frontal and temporal lobes with a small volume of acute subarachnoid hemorrhage (Figure 1).

He was admitted for observation, but during his short stay, he developed symptoms of acute delirium. At the request of the patient and his caregivers, he was subsequently transferred to our institution for further management. The anatomical distribution of these traumatic lesions directly corresponded with the structural networks governing language and cognition, providing a clear etiology for the patient’s acute delirium and subsequent cognitive-linguistic deficits.

### Clinical findings and timeline

Initial comprehensive neurological and psychiatric examinations upon transfer to our institution revealed severe global aphasia and acute delirium. Furthermore, the detailed neurological examination demonstrated mild quadriparesis, with motor strength decreased to Medical Research Council (MRC) grade 4/5 in all four extremities. He was admitted to the neurosurgery department through the emergency department and received ICU care for 3 days. He was managed conservatively to control intracranial pressure and prevent hemorrhage expansion, achieving sufficient clinical stability. On day 7 after injury, he was transferred to the Department of Rehabilitation Medicine for neurorehabilitation. Bedside language examination at that time showed severe global aphasia.

The clinical course and the interventions are summarized in Table 1.

**Table 1:** Timeline of clinical events and interventions

Time point	Clinical, imaging and laboratory findings	Therapeutic strategy
**Day -1**	Reported to work as usual; found collapsed at workplace around lunchtime (heteroanamnesis). CT at outside hospital showed hemorrhagic contusion in the left frontal and temporal lobes with a small amount of acute subarachnoid hemorrhage.	Admission to outside hospital for observation
**Day 0**	Development of acute delirium; Transfered to our institution at patient and caregiver request. Neurological exam revealed severe aphasia, delirium, and quadriparesis (MRC grade 4).	Admission to our NICU; Conservative ICP management
**Day 7**	K-WAB AQ 66.2 (global aphasia); Clinical stability achieved	Transferred to rehabilitation medicine
**Days 7–27**	Resolving hemorrhagic contusions confirmed on follow-up imaging	Cerebrolysin 30 mL/day IV + daily speech-language therapy
**Day 28**	Repeat K-WAB AQ 97.1; near-complete functional language recovery	Continuation of standard neurorehabilitation

### Diagnostic assessment

Formal language testing on day 7 using the Korean version of the Western Aphasia Battery (K-WAB) gave an Aphasia Quotient (AQ) of 66.2 (scale 0–100; higher scores indicate better language function), consistent with severe global aphasia. Follow-up CT demonstrated resolving hemorrhagic contusions in the left frontal and temporal regions without new lesions or complications. The anatomical distribution corresponded well with the patient’s expressive and receptive language deficits.

### Therapeutic intervention

From day 7 after injury, the patient received intravenous Cerebrolysin (EVER Neuro Pharma GmbH, Unterach, Austria) 30 mL/day, diluted in 100 mL of 0.9% sodium chloride and infused over 60 minutes, for 21 consecutive days. Treatment was combined with daily speech-language therapy targeting comprehension, naming, repetition, spontaneous speech, and functional communication. Acute TBI intrinsically carries a higher risk of epileptic seizures due to direct cortical injury and perilesional edema. While there are theoretical concerns about the seizure-threshold-lowering effects of certain neurostimulating agents, Cerebrolysin was administered under close observation. The patient tolerated the treatment exceptionally well, and no clinical seizure activity, infusion reactions, neurological worsening, or other adverse events were observed during the treatment period

### Follow-up and outcomes

**Figure 2:**
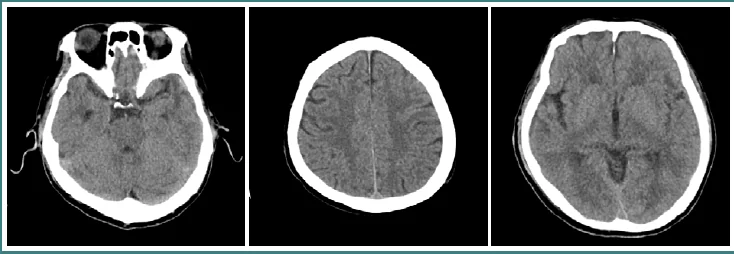
Follow-up brain CT (on day 28 after injury), obtained during rehabilitation, demonstrated progressive resorption. Brain CT showed decreased attenuation of hemorrhagic contusions in the left frontal and temporal lobes.

On day 28 after TBI onset, repeat K-WAB assessment showed dramatic improvement, with the AQ increasing from 66.2 to 97.1. The patient demonstrated near-complete restoration of expressive and receptive language abilities, with fluent functional communication in daily interactions. Although spontaneous biological recovery and the effects of intensive SLT must be considered, the degree of language recovery far exceeded what is commonly expected over a short subacute interval in patients with initially severe aphasia after traumatic hemorrhagic injury. Follow-up brain CT showed decreased attenuation of the hemorrhagic contusions in the left frontal and temporal lobes (Figure 2).

## Discussion

This case demonstrates remarkable short-term recovery from post-traumatic hemorrhagic aphasia following combined Cerebrolysin and speech-language therapy. The temporal association between treatment initiation and substantial improvement in K-WAB scores suggests that Cerebrolysin may have contributed to accelerated language recovery.

Several mechanisms could underlie such an association. Treatment with Cerebrolysin has been reported to promote neuroplasticity through synaptogenesis, dendritic sprouting, and neuronal survival, and to modulate neurotrophic signaling pathways [8]. In hemorrhagic injury, such effects may be particularly relevant in perihematomal and diaschitic cortical regions, where viable but functionally suppressed networks can potentially be re-engaged during rehabilitation.

Clinical evidence for Cerebrolysin is strongest in ischemic stroke, including randomized trials showing improved motor and cognitive outcomes when combined with rehabilitation [9,10]. Emerging preclinical and translational studies also suggest benefits in hemorrhagic injury and traumatic hemorrhage models, including enhanced neuroplasticity and functional restoration [4,5]. Initial RCTs based on these findings have shown evidence for positive clinical effects of Cerebrolysin on cognitive parameters after TBI [11]. The present case raises the possibility that these benefits may extend to language-domain recovery.

The main limitation is the single-patient design, which precludes causal inference. The concurrent use of daily SLT is an important confounder, as speech-language intervention remains the standard of care and likely contributed substantially to recovery. In addition, spontaneous neurological improvement during the subacute phase is expected. Nevertheless, the magnitude of AQ improvement from 66.2 to 97.1 over 21 days is notable and well beyond what is typically expected in an early rehabilitation timeframe, supporting further prospective investigation.

### Patient perspective

The patient’s family reported profound relief following the rapid recovery of language. Initially, the severe aphasia and acute delirium caused immense distress and communication barriers. However, the visible daily improvement during the 21-day Cerebrolysin infusion and concurrent speech therapy provided great reassurance. The patient expressed satisfaction at regaining the ability to communicate effectively with his family and medical staff, noting no specific discomfort or adverse effects during inpatient rehabilitation.

## Conclusion

This case suggests that Cerebrolysin may provide additive benefit when used alongside conventional speech-language therapy in aphasia rehabilitation after traumatic intracranial and subarachnoid hemorrhage. Given the limited pharmacologic options available for traumatic hemorrhage recovery, larger controlled studies are warranted to evaluate efficacy, optimal timing, and safety in language rehabilitation.
